# Molecular characterization and immune functional analysis of IRF2 in common carp (*Cyprinus carpio* L.): different regulatory role in the IFN and NF-κB signalling pathway

**DOI:** 10.1186/s12917-021-03012-7

**Published:** 2021-09-09

**Authors:** Hua Li, Xinping Chen, Yaoyao Zhu, Rongrong Liu, Linlin Zheng, Shijuan Shan, Fumiao Zhang, Liguo An, Guiwen Yang

**Affiliations:** 1 Shandong Provincial Key Laboratory of Animal Resistance Biology, College of Life Sciences, Shandong Normal University, No. 88 East Wenhua Road, Jinan, 250014 China; 2 College of Fisheries and Life Science, Hainan Tropical Ocean University, No. 1 Yucai Road, Sanya, 572022 China; 3 Jinan Eco-environmental Monitoring Center of Shandong Province, No. 17199 Lvyou Road, Jinan, 250101 China

**Keywords:** Common carp (*Cyprinus carpio* L.), Interferon regulatory factor 2 (IRF2), Poly (I:C), *Aeromonas hydrophila*, IFN, NF-κB

## Abstract

**Background:**

Interferon regulatory factor 2 (IRF2) is an important transcription factor, which can regulate the IFN response and plays a role in antiviral innate immunity in teleost.

**Results:**

In the present study, the full-length cDNA sequence of IRF2 (*Cc*IRF2) was characterized in common carp (*Cyprinus carpio* L.), which encoded a protein containing a conserved DNA-binding domain (DBD) and an IRF-associated domain (IAD). Phylogenetic analysis showed that *Cc*IRF2 was most closely related with IRF2 of *Ctenopharyngodon idella*. *Cc*IRF2 transcripts were detectable in all examined tissues, with higher expression in the gills, spleen and brain. *Cc*IRF2 expression was upregulated in immune-related tissues of common carp upon polyinosinic:polycytidylic acid (poly (I:C)) and *Aeromonas hydrophila* stimulation and induced by poly (I:C), lipopolysaccharide (LPS), peptidoglycan (PGN) and flagellin in the peripheral blood leucocytes (PBLs) and head kidney leukocytes (HKLs). In addition, overexpression of *Cc*IRF2 decreased the expression of IFN and IFN-stimulated genes (ISGs), and a dual-luciferase reporter assay revealed that *Cc*IRF2 could increase the activation of NF-κB.

**Conclusions:**

These results indicate that *Cc*IRF2 participates in antiviral and antibacterial immune response and negatively regulates the IFN response, which provide a new insight into the regulation of IFN system in common carp, and are helpful for the prevention and control of infectious diseases in carp farming.

## Background

Interferon (IFN) regulatory factors (IRFs) are key transcription factors in vertebrates and invertebrates, which regulate the expression of IFNs and IFN-stimulated genes (ISGs), and perform diverse biological functions in innate and adaptive immunity [1]. All IRFs contain a highly conserved DNA-binding domain (DBD) at their N-terminus, which is responsible for binding the IFN-stimulated response element (ISRE) in target genes by a helix-turn-helix motif [2]. At the C-terminus is an IRF association domain (IAD), which mediates interactions with several transcription factors and cofactors to form transcriptional complexes [3, 4].

To date, 11 members of the IRF family have been characterized in fish [5], and interferon regulatory factor 2 (IRF2) has been characterized in rainbow trout (*Oncorhynchus mykiss*) [6], mandarin fish (*Siniperca chuatsi*) [7], snakehead (*Channa argus*) [8], zebrafish (*Dario rerio*) [9], stickleback (*Gasterosteus aculeatus*) [9], orange-spotted grouper (*Epinephelus coioides*) [10], Atlantic salmon (*Salmo salar*) [11], paddlefish (*Polyodon spathula*) [12], miiuy croaker (*Miichthys miiuy*) [13], grass carp (*Ctenopharyngodon idella*) [14], blunt snout bream (*Megalobrama amblycephala*) [15], Atlantic cod (*Gadus morhua*) [16] and golden pompano (*Trachinotus ovatus*) [5]. The expression of IRF2 can be upregulated by viral [5–8, 10, 12–14, 16] or bacterial [5, 10, 15] stimulation in different fish tissues, suggesting that IRF2 plays a role in host antiviral and antibacterial responses.

The IFN response is crucial to the antiviral innate immunity of teleost, which can be regulated by IRF2 in different ways. In zebrafish, the expression level of IFNα is increased by the knockdown of IRF2, which has been shown to negatively regulate IFNα signalling [17]. In Atlantic salmon, IRF2 acts as a negative regulatory factor for IFNa1 by competing with IRF-1 [11]. In grass carp, IRF2 can also bind the promoter of IFN via its DBD and downregulate the transcriptional activity of IFN [14]. However, IRF2 plays a positive role in regulating IFNa3 and IFNγ expression in golden pompano [5, 18]. Interestingly, in large yellow croaker, IRF2 was found to induce IFNd and IFNh promoter activity but inhibit IFNc promoter activity [19].

Common carp (*Cyprinus carpio* L.) is a pivotal aquaculture fish species widely cultured in Asia and Europe. Several IRFs have been identified from common carp, including IRF1, IRF3, IRF5, IRF7, IRF9 and IRF10 [20–24]. *Aeromonas hydrophila* is a well-known fish pathogenic bacterium that can cause infection in a number of fish species [25], including common carp (*Cyprinus carpio*) [26], goldfish (*Carassius auratus auratus*) [27], yellow catfish (*Pelteobagrus fulvidraco*) [28] and channel catfish (*Ictalurus punctatus*) [29]. Furthermore, fish are becoming more and more susceptible to *A. hydrophila* because of the increasing intensive rearing methods used in the aquaculture [30]. The previous studies have reported that carp IRFs participated in the immune response against *A. hydrophila* [23]. Thus, in the present study, we identified the full-length cDNA sequence of IRF2 (*Cc*IRF2) in common carp, and investigated its responsiveness to viral and bacterial immune stimulation both in vivo and in vitro. Meanwhile, we determined the roles of *Cc*IRF2 in regulating the IFN response and NF-κB signalling pathway. The present study will contribute to understand the innate immune system of fish, and is helpful for the prevention and control of infectious diseases in carp farming.

## Methods

### Fish feeding and experimental challenge

Common carp (about 200 g per fish) used in the study were purchased from a local fish farm in Jinan, China. After maintained in a fish feeding system (Qingdao Aiwen) at 20 °C for at least one week, 50 fish were anesthetized by immersion in a 100 mg/l solution of MS222 (Sigma), and then evenly divided into two groups to injected intraperitoneally with 500 μl of 2.6 mg/ml poly (I:C) solution (Sigma) or 4.0 × 10^8^/ml *Aeromonas hydrophila*, which were inactivated with 0.5% formalin and resuspended in PBS [23, 31, 32]. At 0, 3, 6, 12, 24, 48 and 72 h post-injection (hpi), three fish were anesthetized and the liver, spleen, head kidney, skin, foregut and hindgut were collected.

### Cloning and analysis of the *Cc*IRF2 cDNA

Total RNA was extracted from head kidney of common carp using TRIzol reagent (TIANGEN), and cDNA was synthesized using a FastQuant RT Kit (TIANGEN). A partial sequence of *Cc*IRF2 was amplified by PCR using the primers IRF2-F/IRF2-R (Table 1), which were designed based on the cDNA sequences of several known fish IRF2. Then, the full-length cDNA sequence was obtained by 3′- and 5′-Full RACE (rapid amplification of cDNA ends) method (TaKaRa). The domains of the *Cc*IRF2 protein were analysed using Simple Modular Architecture Research Tool (SMART, http://smart.embl-heidelberg.de), and multiple sequence alignment and phylogenetic analysis was performed using MEGA 6.0.

**Table 1:** Primers used in the present study

Name of primer	Sequence(5′-3′)	GenBank accession No.
*Cc*IRF2-F	CAGATTCCGTGGATGCATG	
*Cc*IRF2-R	GTCTTCATGATGACGCTGG	
*Cc*IRF2 RT-F	TGGAGCAGAGAGCGATACAGACAG	
*Cc*IRF2 RT-R	GCAGTGCCATCCTCACCTTCTTG	
*Cc*IRF2 ORF-F	CCCAAGCTTATGCCGGTGGAGAGAATGCGT	
*Cc*IRF2 ORF-R	TCCCCGCGGGCAAGTCTTGACGGAGGATTTGG	
*Cc*S11-F	CCGTGGGTGACATCGTTACA	AB012087
*Cc*S11-R	TCAGGACATTGAACCTCACTGTCT	
EPC-IFN-F	CGCTAAGGTGGAGGACCAGGTTA	FN178457
EPC-IFN-R	TTAGGTTCCATTGTGCTGCGTTCA	
EPC-PKR-F	TGGAGACTTCGGCCTCGTGACT	KM099176
EPC-PKR-R	TCGCTTGCTCCGGGCTCATGTA	
EPC-Viperin-F	AAGACTTCCTGGACCGCCATAAGA	KM099177
EPC-Viperin-R	CCTCTCGGCAATCCAAGAAGCG	
EPC-ISG15-F	ACAGTCGGTGAACTCAAGCAAGTC	KM099174
EPC-ISG15-R	CGTAACTGCTGAGGCTTCTGGAAT	
EPC-IRF3-F	CGTGTCCACCACATGCTGAAGG	KJ027520
EPC-IRF3-R	ATCCAGAATCCTCCACCAGCTTGT	
EPC-β-actin-F	GCCGTGACCTGACTGACTACCT	KF844250
EPC-β-actin-R	GCCACATAGCAGAGCTTCTCCTTG

**Table 2:** Protein length and GenBank accession numbers of the IRF2 family members

Species	Protein length	GenBank accession No.
*Cyprinus carpio*	334	MW559072
*Ctenopharyngodon idella*	326	AFV99156
*Danio rerio*	314	NP_001307117
*Scophthalmus maximus*	330	AOV86412
*Paralichthys olivaceus*	330	ADZ96216
*Salmo salar*	343	ACI33066
*Xenopus laevis*	347	NP_001088726
*Gallus gallus*	348	NP_990527
*Mus musculus*	349	AAI10667
*Homo sapiens*	349	CAG3335
*Pinctada maculata*	350	AFZ76969
*Branchiostoma belcheri tsingtauense*	338	AJA02097

### Preparation of PBLs and HKLs from common carp

Peripheral blood leucocytes (PBLs) and head kidney leukocytes (HKLs) of common carp were prepared according to previous studies [23, 33]. In brief, peripheral blood and head kidney cell suspensions were loaded onto freshly prepared 34%/51% Percoll (Sigma) density gradients and separated via centrifugation at 650 g for 30 min. The cells were resuspended and cultivated at 25 °C in Leibovitz’s L-15 medium with 10% foetal bovine serum (FBS), 100 UI/ml penicillin and 100 mg/ml streptomycin.

### Overexpression of *Cc*IRF2 in EPC cells

The open reading frame (ORF) of *Cc*IRF2 was amplified by PCR using Phusion High-Fidelity DNA polymerase (PrimeSTAR), and purified fragments were ligated into the pcDNA3.1-EGFP vector and transformed into *E. coli* Top10 cells. The overexpression vector was extracted from positive clone using an endotoxin-free plasmid isolation kit (TIANGEN) and verified by sequencing. Epithelioma papulosum cyprini (EPC) cells were cultivated at 25 °C in M199 medium (HyClone) with 10% FBS, 100 U/ml penicillin and 100 μg/ml streptomycin (Gibco). Cells were transfected with pcDNA3.1-EGFP-*Cc*IRF2 using X-tremeGENE HP DNA Transfection Reagent (Roche) at 2 μl/well following the manufacturer’s instructions.

### Real-time PCR analysis

Real-time PCR was performed with TransStart Tip Green qPCR SuperMix (TransGen) in a Rotor-Gene Q PCR instrument (Qiagen) [23]. The expression levels of all genes were calculated relative to those of the 40S ribosomal protein S11 or the β-actin gene using the 2^(−∆∆Ct)^ method. The primers used are listed in Table 1.

### Dual-luciferase reporter assays

The effects of *Cc*IRF2 on the activation of NF-κB were performed using Dual-luciferase reporter assays. The 293 T cells were cultivated at 37 °C in DMEM medium (HyClone) with 10% FBS, 100 U/ml penicillin and 100 μg/ml streptomycin (Gibco), and transfected with reporter gene plasmids, pGL-NF-κB-luc and pGL-Renilla-luc, and the pcDNA3.1-EGFP-*Cc*IRF2 vector using Lipofectamine 2000 (Invitrogen). 48 h after transfection, the Dual-Glo® Luciferase Reagent (Promega) was used to measure firefly and Renilla luciferase activity according to the manufacturer’s instructions.

### Statistical analysis

Differences significance analysis were performed using one-way analysis of variance (ANOVA) or T-test in GraphPad Prism 6. All the data were homogeneous and normal, and *P* < 0.05 was considered as significative.

## Results

### Cloning and characterization of *Cc*IRF2

The full-length cDNA of *Cc*IRF2 (GenBank accession No. MW559072) consists of 1916 bp, including an ORF of 1005 bp that translates into a 334-amino acid putative peptide. The 5’and 3′-untranslated region (UTR) of the cDNA is 118 bp and 793 bp, respectively, and an mRNA instability motif (^1861^AATAA^1865^) is contained in the 3′-UTR. The predicted *Cc*IRF2 protein contains a DBD (M1-S115) and an IAD (E211-F265) domain, with six tryptophan residues (Trp11, 26, 38, 46, 58 and 76) and a nuclear localization signal (NLS) in the DBD.

**Fig. 1:**
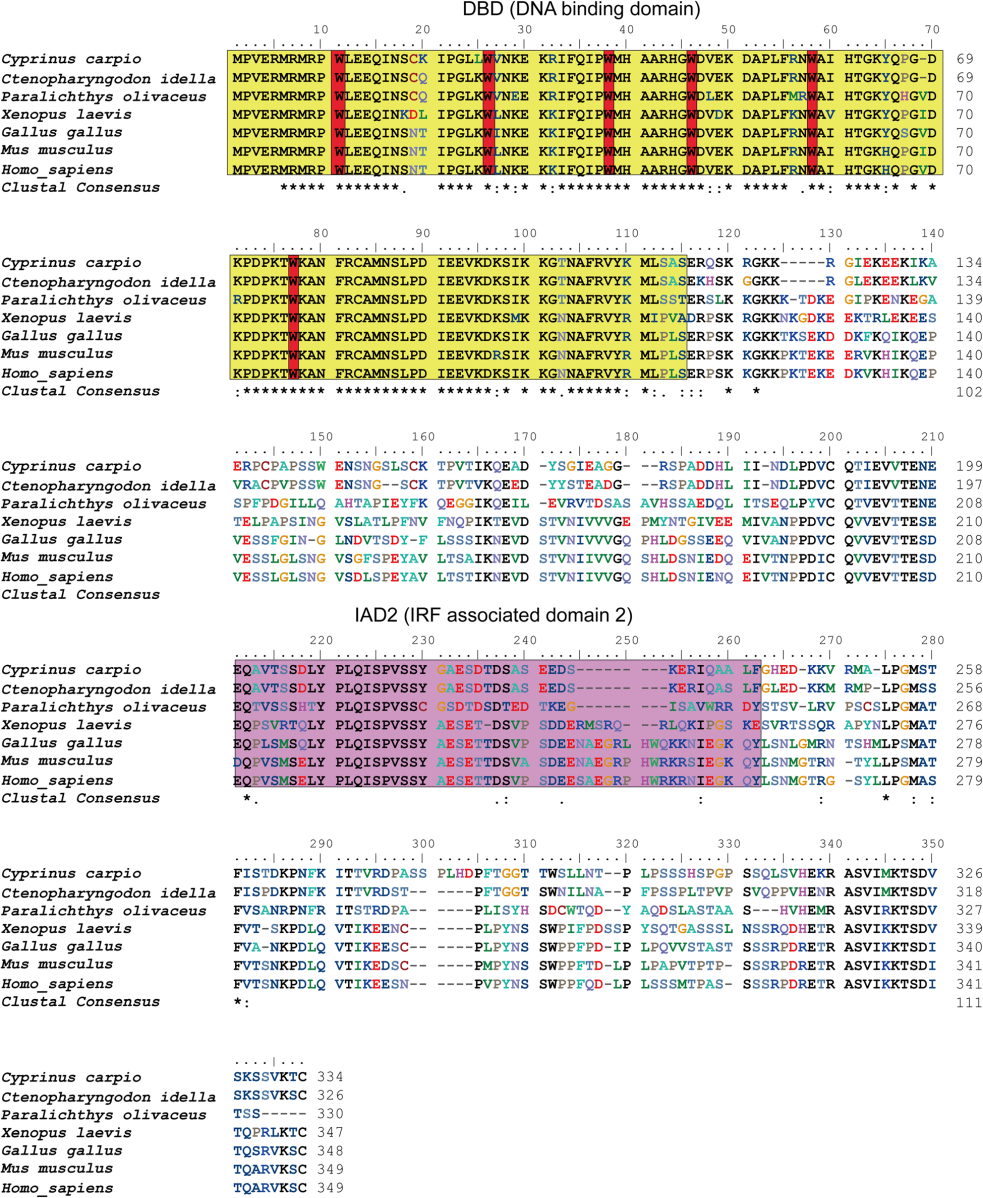
Multiple sequence alignment of IRF2 protein sequences from different species. Identical (*) and similar (: or .) residues are indicated. The DBD and IAD are indicated by black lines. Five tryptophan (W) residues are boxed in red. The GenBank accession numbers of the genes are listed in Table 2

**Fig. 2:**
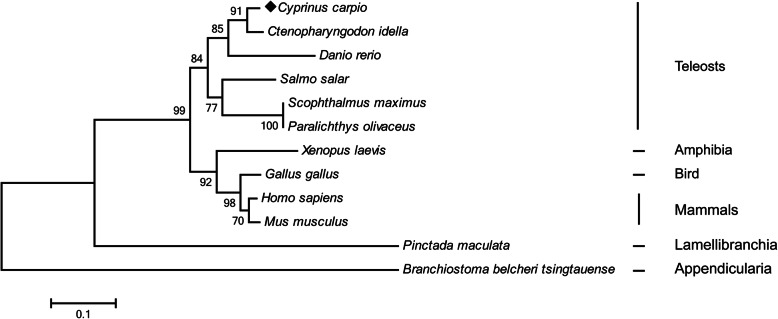
Phylogenetic analysis of IRF2 protein sequences from different species. The phylogenetic tree was produced by the neighbour-joining method in MEGA 6.0. *C. carpio* IRF2 is marked with a solid diamond (◆)

Multiple sequence alignment revealed that IRF2 amino acids sequences were conserved in all vertebrates, and significant homology was found in DBD (Fig. 1). The phylogenetic tree including IRF2 sequences from all known species was constructed using the neighbour-joining method, which was divided into multiple branches, and *Cc*IRF2 was most closely related with IRF2 of *C. idella* (Fig. 2).

### The expression pattern of *Cc*IRF2

**Fig. 3:**
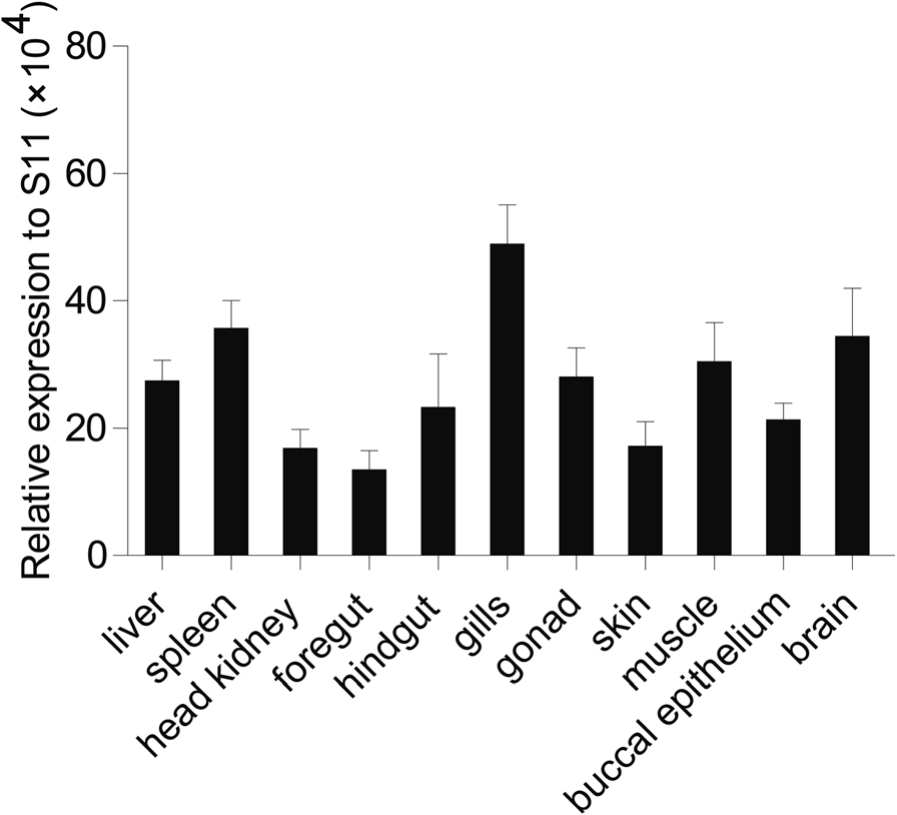
Tissue expression pattern of *Cc*IRF2 under normal physiological conditions. *Cc*IRF2 mRNA expression in the liver, spleen, head kidney, gills, skin, foregut, hindgut, buccal epithelium, gonad, muscle and brain was determined by real-time PCR. Gene expression levels were normalized using the 40S ribosomal protein S11 mRNA (*n* = 3)

The expression of *Cc*IRF2 in eleven tissues of healthy common carp was examined using Real-time PCR method, including the liver, spleen, head kidney, foregut, hindgut, gills, gonad, skin, muscle, buccal epithelium and brain. The results showed that *Cc*IRF2 was expressed in all examined tissues, with higher expression in the gills, spleen and brain (Fig. 3).

### *Cc*IRF2 expression in response to poly (I:C) and *A. hydrophila* stimulation in vivo

**Fig. 4:**
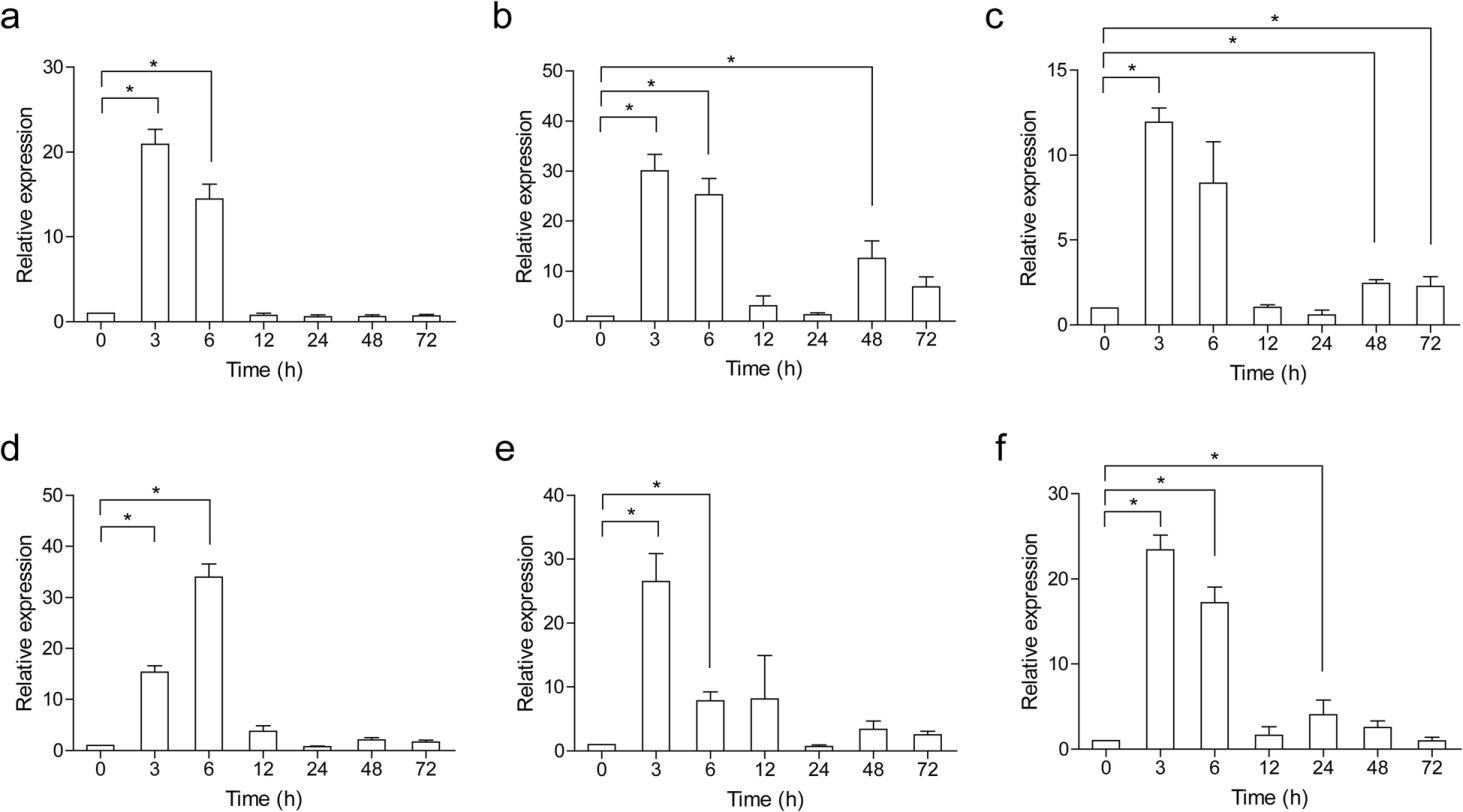
Expression analysis of *Cc*IRF2 in response to poly (I:C) challenge in vivo. Total RNA was extracted from the liver (**a**), spleen (**b**), head kidney (**c**), skin (**d**), foregut (**e**) and hindgut (**f**) at 0 (as a control), 3, 6, 12, 24, 48 and 72 hpi for real-time PCR. Expression was normalized using the 40S ribosomal protein S11 (n = 3, mean ± SD, *P < 0.05)

**Fig. 5:**
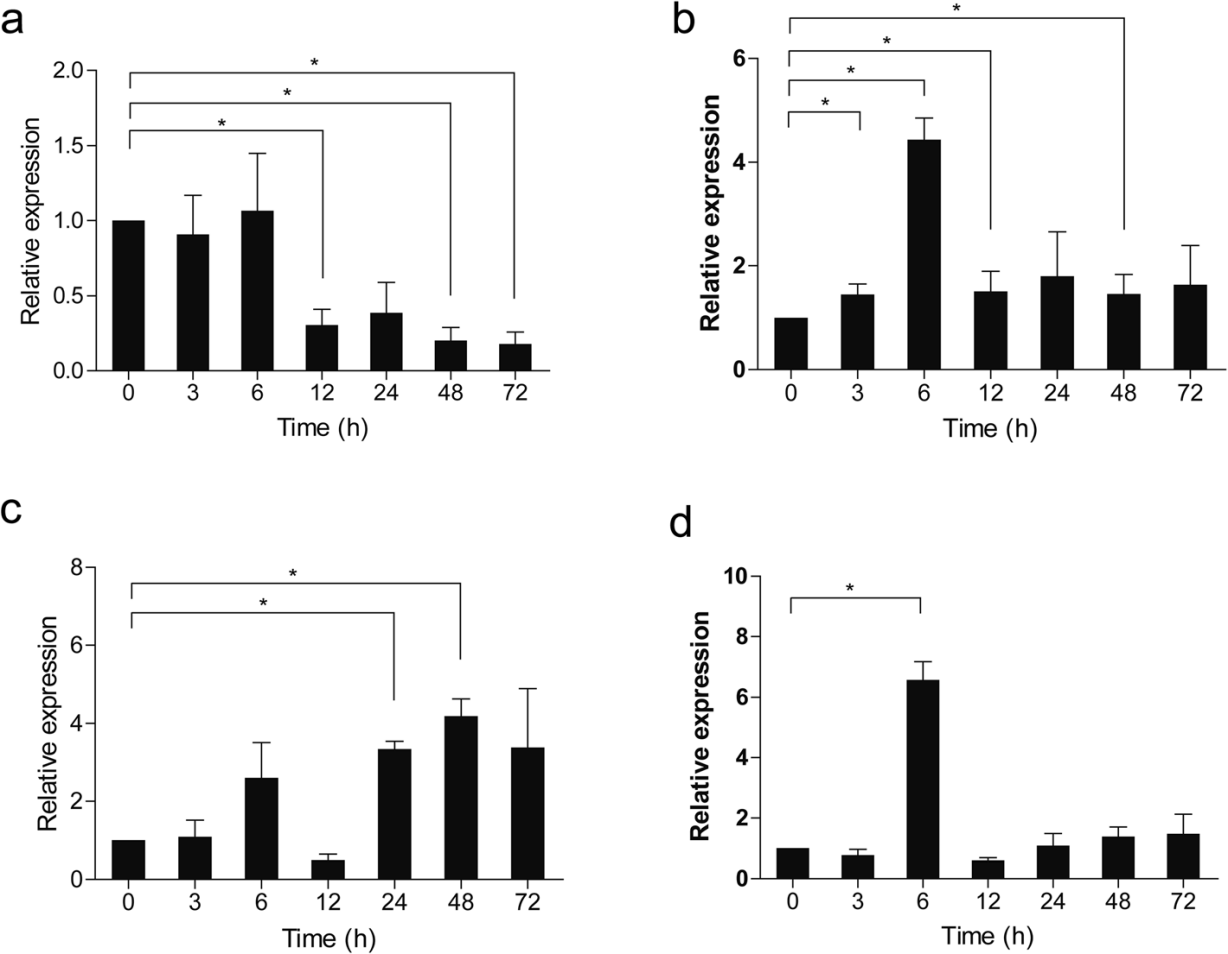
Expression analysis of *Cc*IRF2 in response to *A. hydrophila* challenge in vivo. Total RNA was extracted from the spleen (**a**), head kidney (**b**), foregut (**c**) and hindgut (**d**) tissues at 0 (as a control), 3, 6, 12, 24, 48 and 72 hpi for real-time PCR. The expression was normalized using that of the 40S ribosomal protein S11 (n = 3, mean ± SD, *P < 0.05)

To determine the immune function of *Cc*IRF2, *Cc*IRF2 expression was examined in several immune-related tissues of common carp after viral or bacterial challenge. Upon poly (I:C) stimulation, the expression of *Cc*IRF2 was increased and peaked at 3 hpi in the liver (21.5-fold), spleen (29.7-fold), head kidney (12.4-fold), foregut (26.3-fold) and hindgut (23.4-fold), and peaked at 6 hpi in the skin (34.2-fold) (Fig. 4). In the fish treated with formalin-killed *A. hydrophila*, *Cc*IRF2 expression was upregulated in the head kidney (4.5-fold) and hindgut (6.5-fold) at 6 hpi and in the foregut (13.7-fold) at 24 hpi, while decreased in the spleen at 12 hpi (Fig. 5).

### Expression of *Cc*IRF2 upon poly (I:C), LPS and PGN stimulation in vitro

**Fig. 6:**
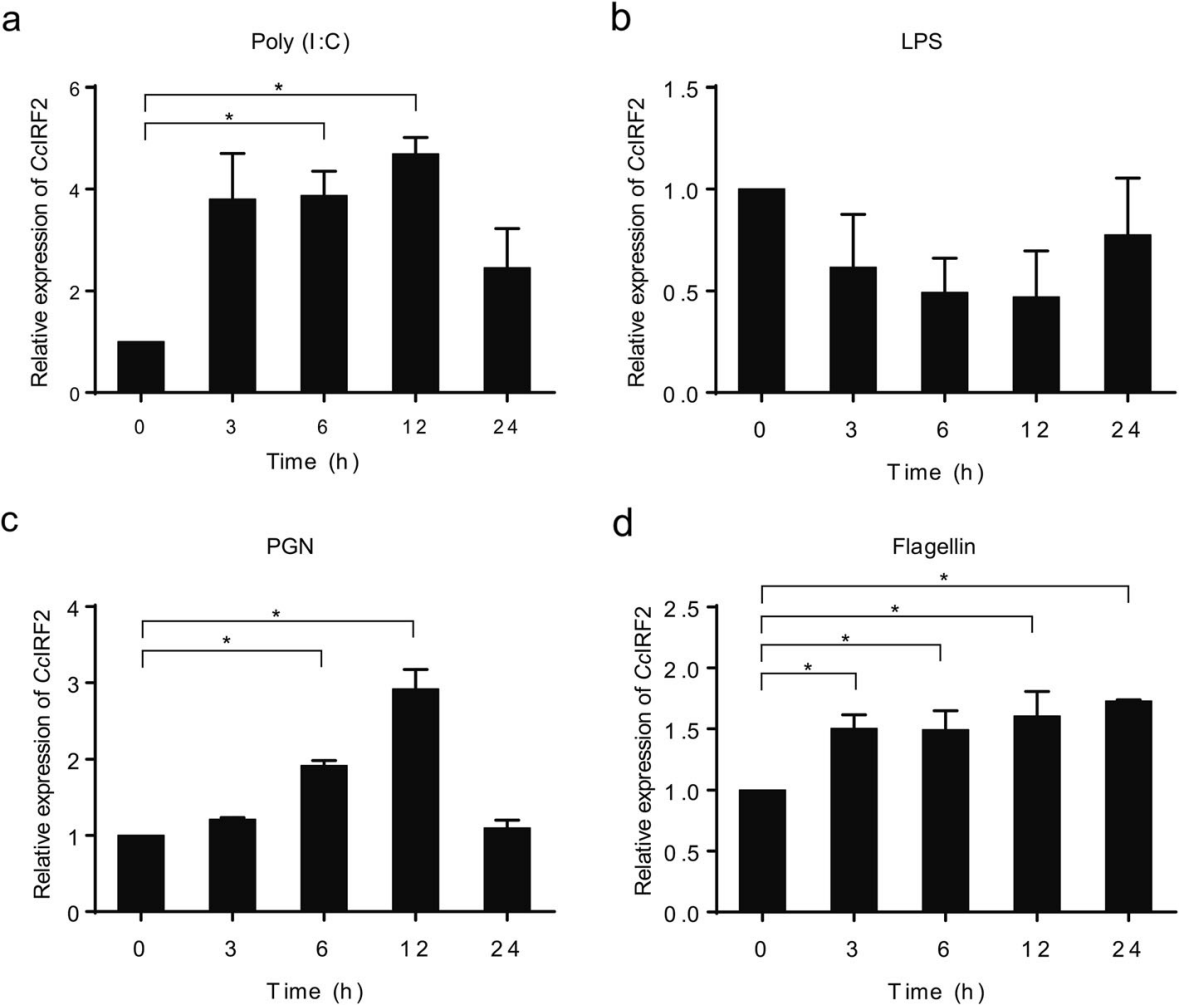
Expression levels of *Cc*IRF2 in PBLs upon different stimulation. Expression of *Cc*IRF2 in the PBLs induced by poly (I:C) (**a**), LPS (**b**), PGN (**c**) or Flagellin (**d**). The expression levels were normalized using the 40S ribosomal protein S11 (mean ± SD, *P < 0.05)

**Fig. 7:**
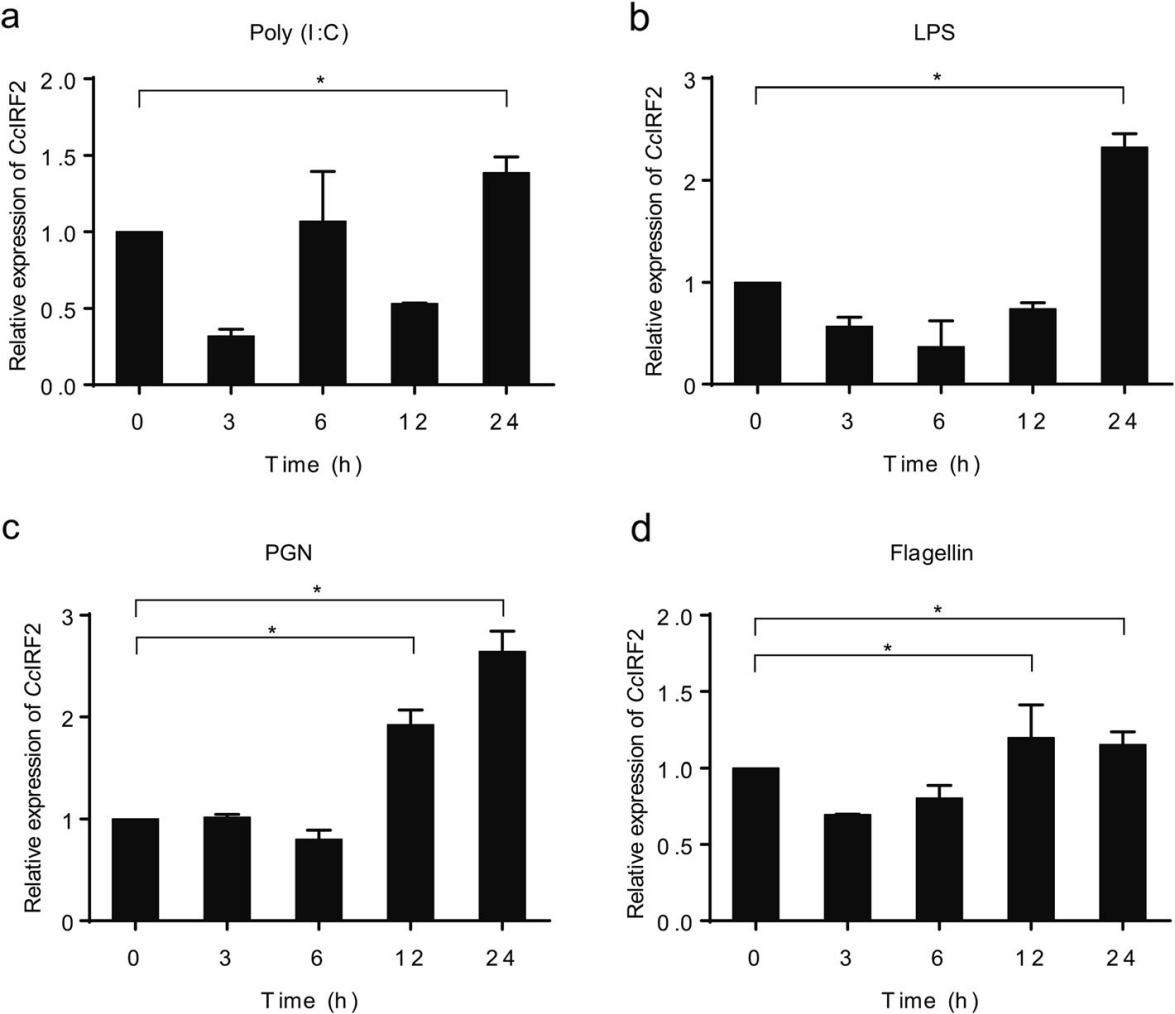
Expression levels of *Cc*IRF2 in HKLs upon different stimulation. Expression of *Cc*IRF2 in the HKLs induced by poly (I:C) (**a**), LPS (**b**), PGN (**c**) or Flagellin (**d**). The expression levels were normalized using the 40S ribosomal protein S11 (mean ± SD, *P < 0.05)

To determine the pathogen-associated molecular patterns (PAMPs) recognized by *Cc*IRF2, the PBLs and HKLs of common carp were stimulated with poly (I:C), LPS, PGN or flagellin, and the expression of *Cc*IRF2 was detected by Real-time PCR. The results showed that *Cc*IRF2 expression in the PBLs was upregulated by poly (I:C) (4.7-fold), PGN (2.9-fold) and flagellin (1.6-fold) at 12 h but not changed by LPS (Fig. 6). In HKLs, *Cc*IRF2 was induced by poly (I:C), LPS, PGN at 24 h (1.4-, 2.4-, and 2.6-fold) and flagellin at 12 h (1.2-fold) (Fig. 7).

### Regulatory role of *Cc*IRF2 in the IFN signalling

**Fig. 8:**
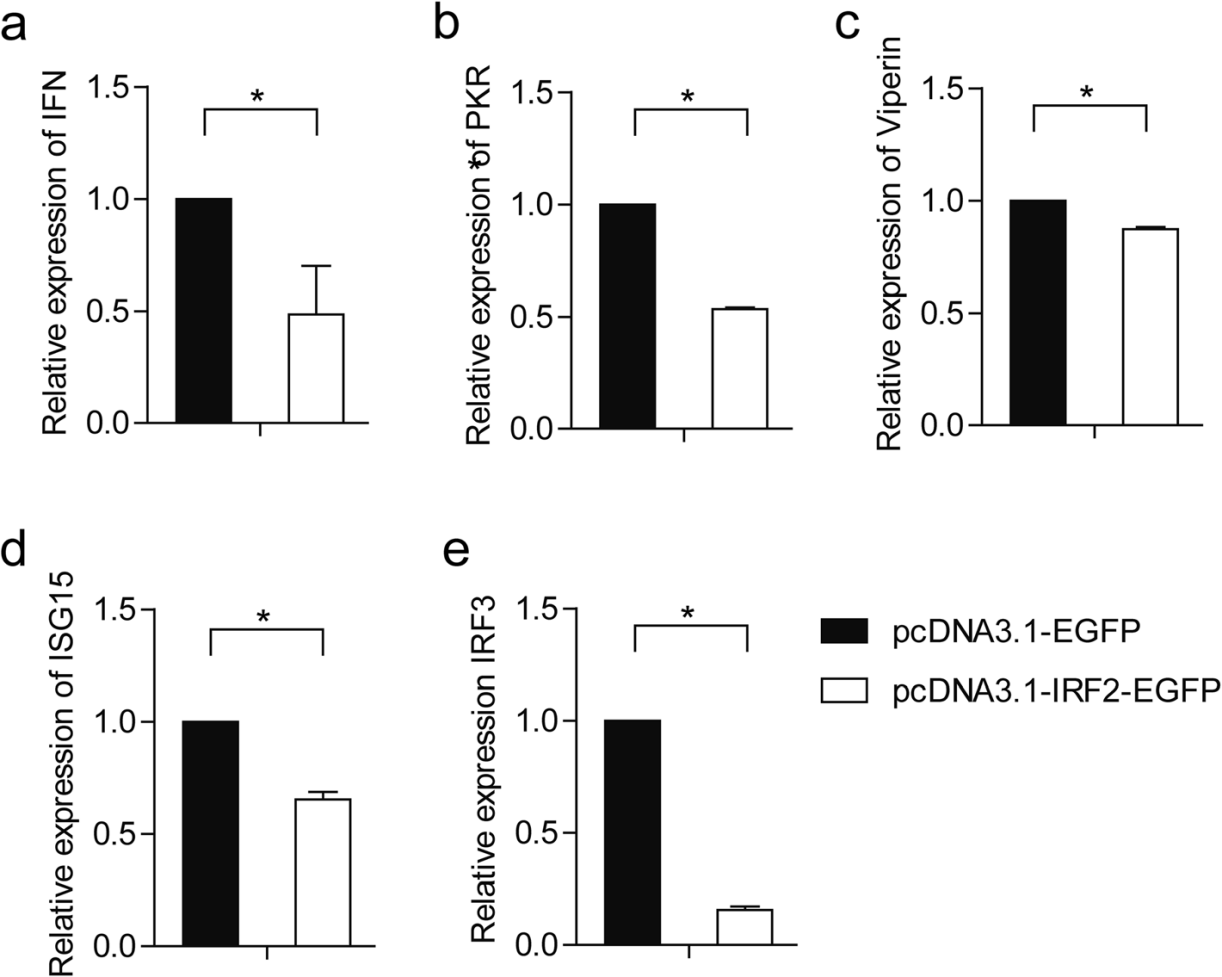
Effect of *Cc*IRF2 overexpression on expression of the IFN (**a**), PKR (**b**), Viperin (**c**), ISG15 (**d**) and IRF3 (**e**) genes in EPC cells. The expression levels were normalized to β-actin (mean ± SD, *P < 0.05)

To investigate the role of *Cc*IRF2 in the IFN signalling pathway, the gene expression of IFN, three ISGs (PKR, Viperin and ISG15) and IRF3 was detected after overexpression of *Cc*IRF2 in EPC cells. The results showed that the expression level of these genes was significantly reduced, with 49, 54, 86, 66 and 15% of the level in control cells, respectively (Fig. 8).

### Regulation of NF-κB by *Cc*IRF2

**Fig. 9:**
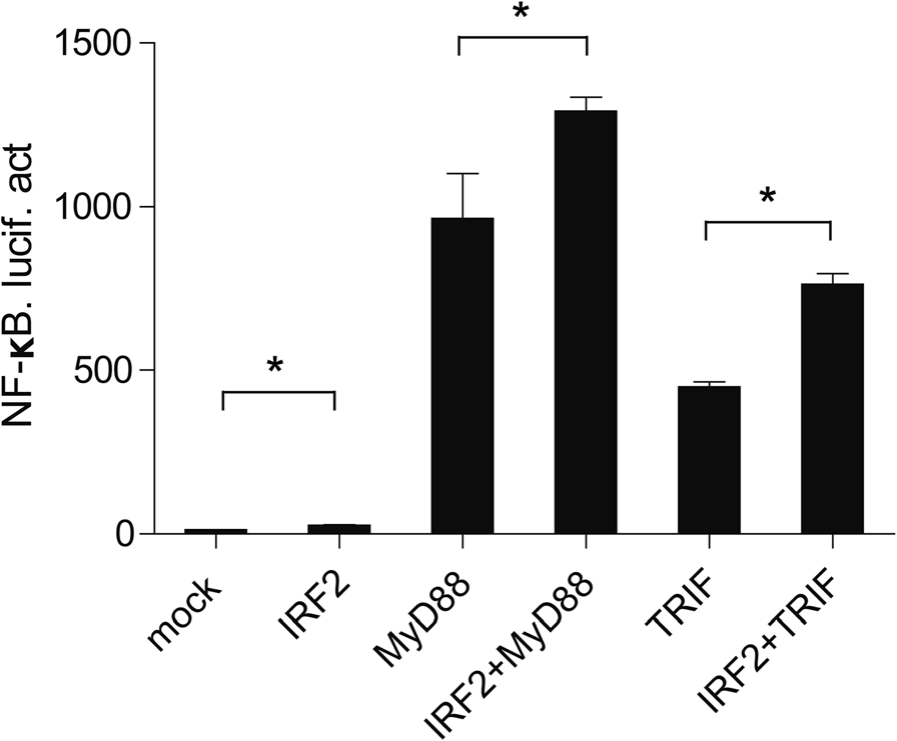
IRF2 positively regulates the NF-κB pathway through MyD88 and TRIF. 293 T cells were cotransfected with the NF-κB reporter gene and *Cc*IRF2 and MyD88/TRIF expression plasmids. Luciferase activity was measured after 48 h and determined against Renilla luciferase activity (mean ± SD, *P < 0.05)

To determine the role of *Cc*IRF2 in the activation of NF-κB, dual-luciferase reporter assay was performed in 293 T cells. The results showed that MyD88 or TRIF could activate NF-κB, and *Cc*IRF2 enhanced MyD88- or TRIF-induced NF-κB activity (Fig. 9).

## Discussion

IRFs, which were originally described as transcription factors induced by IFN, play a variety of roles in immune response, immune system development and cell growth [4, 34–36]. With the development of the fishery economy, an increasing number of studies on IRFs in bony fish have been carried out. In the present study, the full-length *Cc*IRF2 cDNA was cloned from common carp, which encodes 334 amino acid residues. Notably, IRF2 encodes 349 amino acid residues in human and mouse, 348 in chicken and 347 in xenopus, but fewer than 345 in fish, reflecting the important role of IRF2 in the process of biological evolution and possible differences in protein function of IRF2 from different species.

Both mammalian and fish IRFs contain a DBD in the N-terminal region, which is responsible for binding the same ISRE/IRF-E sequences on target genes [37]. The *Cc*IRF2 protein sequence was compared with that from pearl oyster, amphioxus, African clawed frog, chicken, mouse and human. The results showed that the DBD was relatively conserved, and the DBD in all species except amphioxus contained six tryptophan residues, suggesting that the function of the IRF2 molecule may be relatively evolutionarily conserved. There are two types of IRF C-termini: IAD1 was originally found in IRF8 and is present in all members of the IRF family except IRF1 and IRF2, while IAD2 exists in only IRF1 and IRF2 [38]. In the present study, the C-terminus of *Cc*IRF2 contains the IAD2 domain.

The known IRF2 protein sequences from different species were used to construct an evolutionary tree using the neighbour-joining method. In the tree, IRF2s of teleosts, amphibia, bird and mammals are on a branch, while IRF2s of lamellibranchia and appendicularia are separate. The 11 members of the fish IRFs family are divided into four clades: the IRF1 subfamily, consisting of IRF1, IRF2 and IRF11; the IRF3 subfamily, consisting of IRF3 and IRF7; the IRF4 subfamily, consisting of IRF4, IRF8, IRF9 and IRF10; and the IRF5 subfamily, consisting of IRF5 and IRF6. *Cc*IRF2 is closely related to grass carp in the branch containing fish species, which belongs to the IRF1 subfamily.

IRF2 is widely expressed in a variety of tissues in bony fish, such as *E. coioides* and *O. mykiss* [6, 10]. In the present study, the expression pattern of *Cc*IRF2 in 11 tissues of healthy common carp was investigated, which was expressed in all tissues, with higher expression level in the gills, spleen and brain. Concordant with these results, the expression of IRF2 is highest in the gills or spleen of *P. spathula*, *M. amblycephala*, *C. argus* and *C. idella* [8, 12, 15]. In fish, the spleen and gills are important systemic and mucosal immune organs, respectively, and play a key defensive role against pathogen invasion, suggesting that *Cc*IRF2 plays an important role in the immune system of fish. Unexpectedly, the expression level of IRF2 in the brain of common carp was also high, implying that IRF2 may be involved in regulating the nervous system of fish.

The previous studies in fish have shown that IRF2 can be induced by poly (I:C), including mandarin fish, paddlefish, rainbow trout and snakehead [6–8, 12], and the expression level of IRF2 in the liver, spleen and head kidney of half-smooth tongue sole was also significantly increased after viral stimulation [39]. In the present study, after poly (I:C) stimulation, the expression of *Cc*IRF2 in all tissues increased and peaked at 3 h or 6 h, and the expression of *Cc*IRF2 in the skin and spleen increased to the maximum level, suggesting that IRF2 may play an important role in the early immune response of common carp to viruses. After stimulation by *A. hydrophila*, the expression of *Cc*IRF2 in the head kidney, foregut and hindgut was increased, but significantly decreased in the spleen. Similar to the results, after stimulation with *A. hydrophila*, the expression of IRF2 was increased in the spleen and head kidney but significantly decreased in the gills and intestines of blunt-snout bream. Therefore, the antibacterial immune process in which IRF2 is involved may be tissue-specific.

Similar to the in vivo results, poly (I:C) induced an increase in *Cc*IRF2 expression in PBLs and HKLs. However, there was no significant change in *Cc*IRF2 expression in PBLs after LPS stimulation. Studies in mammals have shown that LPS can induce the expression of IRF genes in B cells, T cells, macrophages and dendritic cells [40, 41]. However, fish can resist the toxic effects of LPS [42], and lack costimulatory molecules (such as MyD2 and CD14) produced by TLR4 during LPS-induced immune activation [43]. Therefore, the unchanged expression of *Cc*IRF2 after LPS stimulation may be due to the different mechanism of LPS recognition between fish and mammals.

Mammalian IRF2 is unrelated to the production of IFN but is involved in the transcriptional regulation of downstream genes such as histone H4, IL-7, IL-12, iNOS and MHC class I molecules [44–48]. The previous studies have shown that mammalian IRF2 plays a role in inhibiting transcriptional regulation due to its competition with IRF1 [49], and amphioxus IRF2 can compete with other IRFs for the ISRE-binding site in the nucleus, thus IRF2 is considered a negative regulator of the transcription process [50]. In this study, the overexpression of IRF2 in common carp inhibited the expression of IFN, PKR, Viperin, ISG15 and IRF3 in EPC cells, so *Cc*IRF2 may also negatively regulate the expression of IFN and related factors. However, the dual-luciferase reporter assay showed that *Cc*IRF2 activated the NF-κB signalling pathway and promoted both MyD88-mediated and TRIF-mediated NF-κB production. Mammalian IRF2 was reported to regulate NF-κB activity by regulating the subcellular localization of NF-κB [51], and IRF2 in pearl oyster can also activate the NF-κB signalling pathways [52]. Thus, IRF2 belongs to a class of multifunctional transcription factors whose specific roles in transcriptional inhibition or activation depend on cell type and inflammatory state [4, 53, 54].

## Conclusions

In the present study, the full-length cDNA of the IRF2 gene was identified from common carp, and its effects on defence against pathogen invasion and regulation of the IFN response were investigated in vivo and in vitro. The results indicate that *Cc*IRF2 participated in antiviral and antibacterial immunity and negatively regulated the IFN response, which provide a new insight into the regulation of IFN system in common carp, and are helpful for the prevention and control of infectious diseases in carp farming.

## Data Availability

The dataset supporting the conclusions of this article is available in the GenBank (https://www.ncbi.nlm.nih.gov/nuccore/MW559072) and the accession number is MW559072.

## Abbreviations

IFN: interferon
IRF: interferon regulatory factor
ISG: IFN-stimulated gene
DBD: DNA-binding domain
NLS: nuclear localization signal
IAD: IRF associated domain
poly (I:C): polyinosinic:polycytidylic acid
LPS: lipopolysaccharide
EPC: epithelioma papulosum cyprinid
RACE: rapid amplification of the cDNA ends
SMART: simple modular architecture research tool
PBL: peripheral blood leucocyte
HKL: head kidney leukocyte
PGN: peptidoglycan
ORF: open reading frame
ANOVA: one-way analysis of variance
UTR: untranslated region
hpi: hour post injection
ISRE: IFN stimulating response element
IRF-E: IRF regulatory element
